# Fragility Assessment of RC Bridges Exposed to Seismic Loads and Corrosion over Time

**DOI:** 10.3390/ma16031100

**Published:** 2023-01-27

**Authors:** Daniel Herrera, Dante Tolentino

**Affiliations:** Departamento de Materiales, Universidad Autónoma Metropolitana, 420 San Pablo Avenue, Nueva el Rosario, Azcapotzalco, Mexico City 02128, Mexico

**Keywords:** cumulative damage, fragility assessment, reinforced concrete bridges, corrosion deterioration, seismic loads

## Abstract

A methodology to estimate the structural fragility of RC bridges, considering the effects of seismic loadings and corrosion over time, is presented. Two scenarios are considered: (a) The structure is exposed only to seismic loads, (b) Both the effect of corrosion and seismic loads are present in the system. The uncertainties related to material properties, structural geometry, seismic occurrences, corrosion initiation time, cracking and corrosion evolution are considered. Different time stages, such as 0, 50, 75, 100, and 125 years are selected to evaluate the effect of both seismic loads and seismic loads plus corrosion. The calculation of fragility curves implies a structural design, nonlinear modeling of structures with simulated properties, estimation of both corrosion times and seismic occurrences, and evaluation of structural demand over time considering the effect of seismic loads and corrosion. An illustrative example is provided on an RC continuous bridge with AASHTO beams, cap beams and circular columns located in Acapulco, Guerrero, Mexico. A performance level equal to 0.002 is chosen for the design of the structure. Results show that the probability of exceeding the design performance levels for both cases (seismic and seismic plus corrosion) are similar at the stage of time equal to zero (a newly built bridge). However, such probabilities, after 150 years, are equal to 0.61 and 0.85 due to the cumulative damage caused by seismic and seismic plus corrosion, respectively. The estimation of the probability of exceeding a certain performance level, considering the effect of corrosion together with seismic loads, highlights the importance of considering more than one type of solicitation for these kinds of structural systems. Lastly, recommendations about design are given.

## 1. Introduction

Bridges are subject to different environmental hazards during their life span. Such hazards include wind, scouring, waves, earthquakes and corrosion. Most bridges in coastal zones are exposed to corrosion and earthquakes. As observed in the recent natural disasters caused by earthquakes such as Japan 2011, Japan 2016, and Mexico City 2017, road networks played a crucial role in both evacuating affected people and transporting emergency materials and equipment, making it essential to keep roads in an acceptable condition. The absence of maintenance in this type of structure leads to a reduction of its capacity to resist subsequent loads, and the probability that the structure exhibits undesired performance levels is increased. Therefore, many researchers have been interested in proposing criteria that allow the evaluation of the probability of exceeding different performance levels. An example is a study that estimates fragility curves in RC bridges considering the effects of spatial variation in the seismic response [1]. Different methodologies have been proposed to estimate fragility curves in RC bridges with the purpose of estimating economic losses [2,3,4,5] and establishing repair activities [6,7]. Fragility curves have been estimated in order to reduce lateral displacements in RC bridges using base isolation [8]. The uncertainties related to both demand and capacity have been considered to calculate fragility curves [9]. Ref. [10] evaluates the risk of highway bridges exposed to seismic events, obtaining fragility curves for different limit states. Ref. [11] evaluates the influence of steel fiber-reinforced concrete on the performance of concrete bridges with respect to traditional reinforcement. The seismic fragility of high-speed railway bridges is estimated, both with and without considering the track system [12]. The effect of earthquake damage on structural fragility over time has been considered [13].

Corrosion in reinforced concrete elements begins when sea breeze transports and deposits chloride ions on them. When a sufficient quantity of those ions accumulates on the surface of such concrete elements, the ions seep through the concrete cover and generate an electrochemical process with the reinforcing steel. Chloride ions are hygroscopic: they are capable of both absorbing and containing moisture, causing the electrical resistance of the concrete to decrease, thus allowing the easy transfer of chloride ions through the pores of the concrete. Thus, several methodologies that consider concrete as a homogenous material to estimate cracking have been presented. Ref. [14] simulates the concrete cracking of two reinforcements, proposing a delimitation for cover thickness and using the finite element method. Ref. [15] presents a three-dimensional FE methodology to assess the cracking in concrete cover due to both uniform and non-uniform corrosion. Ref. [16] proposes a two-dimensional, chemical-mechanical model to predict the concrete cover cracking induced by non-uniform corrosion. The previous numerical models have been improved considering concrete as an anisotropic material: [17] proposes a new phase field method for modeling the material in multiple failures; a new structural tensor that considers the fracture energy is presented. The proposed expression allows both to predict cracking propagation and estimate different failure modes of the material. Ref. [18] propose a mesoscale numerical model to assess both the initiation and the evolution of cracking induced by non-uniform corrosion of multiple reinforcements considering concrete as an anisotropic material. Furthermore, the effect of corrosion on structural performance in a probabilistic way has been studied to estimate the reliability of corroded beams [19,20,21,22,23]. Stochastic models have been proposed to evaluate crack width, the diffusion coefficient and reliability profiles of RC structures under the effect of corrosion [24,25,26]. Moreover, the distribution of corrosion products in both cracked concrete and the alkaline layer in the steel reinforcement has been examined [27,28]. Ref. [29] presents a time-dependent model to simulate the corrosion phenomenon.

Deterioration produced by corrosion plus seismic loads may force the abutment to move either away from or toward the backfill soil of the abutments. Therefore, the consideration of the interaction between superstructure, foundation, abutments and backfill soil in bridge design is important [30,31]. Abutment–backfill soil interaction problems can be avoided by means of a soil reinforcement approach [32,33] or by adding tire rubber to the backfill of bridge abutments [34]. On the other hand, [35] both calculate the fragility curves of corroded concrete bridges and propose seismic loads for the structural reliability assessment due to the effect of corrosion. Refs. [36,37,38] propose a methodology to estimate the failure probability of RC structures due to airborne chloride by considering the aleatory and epistemic uncertainties involved in the corrosion phenomenon. For structures in a moderately to highly aggressive environment, numerous researchers have pointed to theoretical predictions of structural performance over time. Ref. [39] estimates the structural reliability of corroded RC structures under seismic sequences in marine environments. Ref. [40] presents a methodology to assess the effect of corrosion deterioration on the structural reliability of RC buildings. Ref. [41] evaluates the seismic vulnerability of corroded concrete frames considering different seismic damage limit states. Ref. [42] presents a methodology to evaluate the reliability of RC structures using the finite element method. Ref. [43] presents a numerical investigation to assess the influence of corrosion damage on the seismic fragility of RC bridge columns subjected to both static and dynamic loads. Ref. [44] estimates the fragility curves of corroded RC bridge piers using both a fiber-based finite element model and Markov chains. Ref. [45] calculates the failure probability and its corresponding reliability index of corroding concrete bridge girders.

Taking the work of references previously mentioned into account, it is required to propose methodologies to estimate the probability of exceeding a certain threshold considering the effect of seismic loads and seismic loads plus corrosion over time. The results obtained in this paper are used to compare the probability of exceeding a certain performance threshold with the recommendation of the AASHTO design code, which indicates that a bridge structure must remain in service for up to 75 years after its construction.

## 2. Fragility Estimation

Structural reliability methods provide a mathematical model to consider the uncertainty of hazards, structural models, and solicitations, among other variables. The fragility curve is one of the most widely used mathematical tools to represent structural performance in probabilistic terms. Such a curve represents the probability that the structural response exceeds a specific threshold for a given solicitation. Based on the investigations realized by [46], the structural demand can be characterized by a lognormal probability density function. Then, the fragility curve that considers the effect of corrosion can be calculated as follows:

(1) $$P\left(D{\left(t\right)}_{corr}{}_{\left|y\right.}\geq d\right)=1-\int f_{D}\left(d,t\right)dd\;=1-\int \varphi \frac{1}{\sigma_{\ln D{\left(t\right)}_{corr}{}_{\left|y\right.}}d}\left(\frac{\ln\left(d\right)-\ln\left(\mu_{\ln D{\left(t\right)}_{corr}{}_{\left|y\right.}}\right)}{\sigma_{\ln D{\left(t\right)}_{corr}{}_{\left|y\right.}}}\right)dd\;=1-\Phi \left(\frac{\ln\left(d\right)-\ln\left(\mu_{\ln D{\left(t\right)}_{corr}{}_{\left|y\right.}}\right)}{\sigma_{\ln D{\left(t\right)}_{corr}{}_{\left|y\right.}}}\right)$$

where φ represents the Gaussian distribution function; d is a pre-established demand threshold; Φ is the normal cumulative distribution function. The median value of demand for a certain corrosion state, given a seismic intensity, y, at the stage t is equal to $\mu_{\ln D{\left(t\right)}_{corr}{}_{\left|y\right.}}=\exp\left(\overset{n}{\underset{i=1}{\sum }}\ln\left(Di{\left(t\right)}_{corr}{}_{\left|y\right.}\right)/n\right)$ and its standard deviation of the natural logarithm for a certain state of corrosion, given an intensity, y, at the stage t is $\sigma_{\ln D{\left(t\right)}_{corr}{}_{\left|y\right.}}={\left[\overset{n}{\underset{i=1}{\sum }}{\left(\ln\left(Di_{corr}{}_{\left|y\right.}\right)-\ln\left(\mu_{\ln D{\left(t\right)}_{corr}{}_{\left|y\right.}}\right)\right)}^{2}/n-1\right]}^{1/2}$. Parameter $Di{\left(t\right)}_{corr}{}_{\left|y\right.}$ is the natural logarithm of the structural response given an intensity, y, at the stage t, and n is the number of observations. 

## 3. Corrosion Assessment

**Figure 1:**
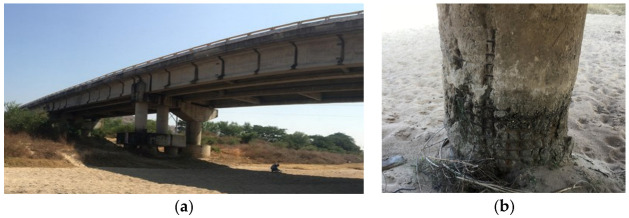
Bridge structure located near to Mexican Pacific coast: (**a**) longitudinal view and (**b**) structural element with deterioration.

The evaluation of corroded structures has attracted the attention of many researchers because corrosion accelerates the vulnerability of bridges exposed to seismic loads, making it essential to evaluate the effect of corrosion in bridges located in highly seismic regions. Scientific evidence shows that steel embedded in concrete has a protective layer due to the calcium carbonate contained in cement [29]. If it is assumed that the quality of the concrete is low, there is a high probability that the structural elements present a critical concentration of chloride ions, which act as corrosion catalysts. Then, the protective layer may disappear, leading to the initiation of corrosion, generating an oxide layer that causes additional internal stresses and cracking of the concrete cover, which significantly affects both the structural performance of the structural elements and the overall safety of the structural system. Structures near the Mexican coast in the Pacific are exposed to corrosion and seismic loads due to their proximity to the Cocos plate. Figure 1 shows a bridge located on the Pacific Coast with corrosion deterioration. An alternative to reinforcing deteriorated structural elements is the use of glass fiber-reinforced polymer (GFRP) [47]. Such material offers advantages such as corrosion resistance, energy dissipation under seismic loads, and low construction cost. 

### 3.1. Corrosion Initiation Time

The penetration of chloride ions into concrete is a highly complex process since it involves different transport mechanisms, for example, ionic diffusion and capillary suction. Furthermore, chloride penetration depends on the composition of the concrete, its degree of saturation, cover thickness, porosity, and exposure conditions. Such a chloride penetration process can be described on the basis of a practical model such as Fick’s second diffusion law [48]. The diffusion coefficient, Ψ, can be characterized by considering the water–cement ratio and the environment temperature, as follows [25]:

(2) $$\Psi =11.146-31.025\left(\frac{w}{c}\right)-1.941\phi +38.212{\left(\frac{w}{c}\right)}^{2}+4.48\left(\frac{w}{c}\right)\phi +0.024\phi^{2}$$

where ϕ is the local temperature, and w/c represents the water–cement ratio. On the other hand, the time that chloride ions take to reach the position of the steel reinforcement is determined by the following expression:

(3) $$T_{corr}=\frac{d_{0}^{2}}{4\Psi }{\left[erf^{-1}\left(\frac{C_{cr}-C_{0}}{C_{i}-C_{0}}\right)\right]}^{-2}$$

where $T_{corr}$ is the corrosion initiation time; $C_{cr}$ is the critical ion concentration; $C_{i}$ is the initial chloride concentration; $C_{0}$ is the equilibrium concentration of chlorides on the concrete surface as % of the cement weight; $d_{0}$ represents the concrete cover. 

### 3.2. Corrosion Evolution

The evolution assessment of corrosion in concrete can be calculated by a linear relationship between the diameter of the steel and time as follows [26]:

(4) $$H\left(t\right)=\eta_{0}-i_{corr}\left(t-T_{corr}\right)c_{corr}$$

where $H\left(t\right)$ is the reduced diameter at time t; $\eta_{0}$ is the initial diameter; $c_{corr}$ is the corrosion coefficient and $i_{corr}$ represents the corrosion rate. Ref. [49] performs a sensitivity analysis to estimate the reduction of flexural strength of beams with corroded bars due to different area reductions using Equations (3) and (4). The numerical results are compared with experimental data provided by [50,51,52]. Maximum differences of 27% are found. Once the deterioration of the cross-sectional area begins, an oxide layer is generated around the steel reinforcement. Then, additional internal stresses in the structural elements are generated. Ref. [24] proposes an expression to determine the number of corrosion products capable of filling the pores in concrete as follows:

(5) $$W_{pore}=\pi t_{pore}\rho_{rust}\eta_{0}$$

where $W_{pore}$ is the volume of oxide required to fill a pore; $t_{pore}$ is the thickness in the zone equivalent to a porosity of 1 and $\rho_{rust}$ is the density of the oxide.

### 3.3. Cracking Initiation Time

The corrosion products, $W_{crit}$, involved in the initial cracking of concrete consist of three volumes: (1) the porous zone, $W_{pore}$, (2) the amount of rust that may induce internal pressure in concrete, $W_{expan}$ and (3) the space of the corroded steel, $W_{steel}$. The corrosion products, $W_{crit}$, are estimated as follows [26]:

(6) $$W_{crit}=W_{steel}+W_{pore}+W_{expan}$$

(7) $$W_{expan}=\pi \rho_{\mathrm{rust}}\left(\eta_{0}+2t_{pore}\right)\frac{df\prime_{t}}{E}\left(\frac{a^{2}+b^{2}}{b^{2}-a^{2}}b+v_{c}\right)$$

(8) $$W_{steel}=\frac{\rho_{steel}}{\rho_{steel}-\alpha \rho_{\mathrm{rust}}}$$

where $f\prime_{t}$ is the tensile stress of the concrete; E is the modulus of elasticity of the concrete; $v_{c}$ is the Poisson ratio; the inner radius of idealization is $a=\eta_{0}+2t_{pore}/2,$ and the outer radius of idealization is $b=d+\eta_{0}+2t_{pore}/2\;$; $\rho_{steel}$ is the density of steel reinforcement; α is a constant related to corrosion products [53]. Ref. [24] proposes an expression based on experimental studies of [53] to estimate the cracking initiation time as follows:

(9) $$\Delta t_{crack}=\frac{{\left(W_{crit}\right)}^{2}}{2\left[\left(0.383\times 10^{-3}\right)\eta_{0}i_{corr}\right]}$$

## 4. Cumulative Damage Estimation 

The action of seismic loadings on a structure during a time interval may cause damage to the structural elements. On the other hand, considering the dominant hazard site does not guarantee that bridges present adequate performance levels when another environmental load, such as corrosion, appears. Thus, quantifying the potential cumulative damage caused by one or more environmental loads is of prime importance. If it is assumed that during the time interval in question, there is no repair or maintenance in the system, the possible cumulative damage is given as in Algorithm 1.
**Algorithm 1** The pseudocode of cumulative damage1:  Begin 2:  n bridges with uncertain properties are generated 3:  Realizations of seismic occurrences associated with each bridge model are generated 4:  Time thresholds of interest, m, associated with corrosion are estimated 5:  Different time stages, T, are selected 6:  Initialize counters i=1, k=1 and $t_{0}=0$ 7:  while k≤n
8:   while i≤m
9:    $t=t_{0}+\Delta t_{i+1}$ 10:   while t≤T
11:      if i=1
12:      The i-th and $\left(i+1\right)-\mathrm{th}$ intensities are associated with the k-th structural model 13:       Two seismic records are associated with the i-th and $\left(i+1\right)-\mathrm{th}$ intensities 14:       Each record is modified by a factor $\psi_{e}=i_{sim}/i_{T}$ that relates the intensity and the value of spectral acceleration at the fundamental period of the k-th system 15:       $Di_{corr}{}_{\left|y,t\right.}$ of the k-th system is calculated 16:       A random ground motion, $S_{ki}$, is modified by a factor, βm, that matches $Di_{corr}{}_{\left|y,t\right.}$ 17:       else 18:       A random seismic record, $r_{i}$, is associated with the $\left(i+2\right)-\mathrm{th}$ simulated intensity and is scaled by the factor $\psi_{e}$ 19:       The system is subjected to a seismic signal composed of the seismic record, $S_{ki}$, and the seismic record, ri 20:       $Di_{corr}{}_{\left|y,t\right.}$ of the k-th system is calculated 21:       A ground motion, $S_{k\left(i+1\right)}$, is selected randomly, and it is modified by a factor, βm, that matches $Di_{corr}{}_{\left|y,t\right.}$ 22:       A reduction of the cross-sectional area of the reinforcement steel is performed 23:       The ground motion $S_{k\left(i+1\right)}$ at the stage t is scaled up until the structure fails 24:    add one to the intensities counter 25:   add one to the simulated bridges counter 26:  end 

## 5. Illustrative Example

**Figure 2:**
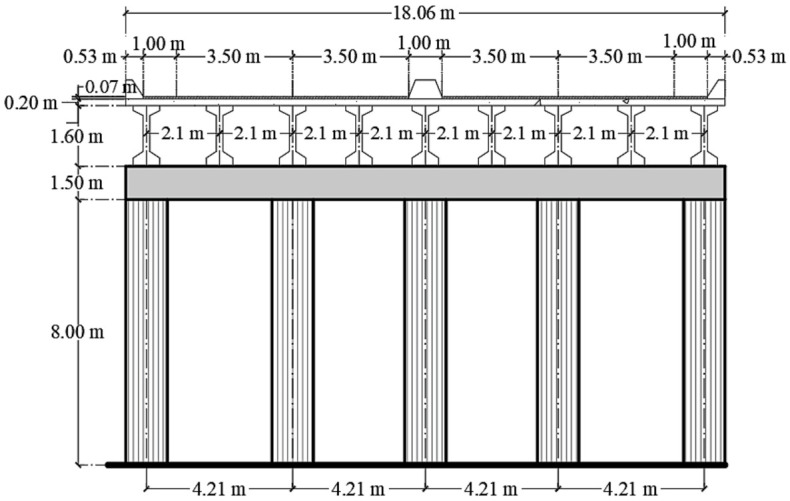
Transverse section.

**Figure 3:**
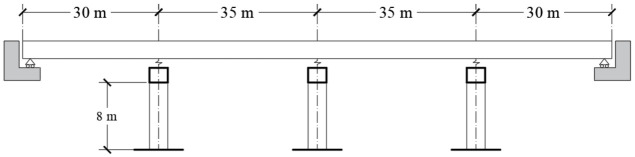
Longitudinal section.

**Figure 4:**
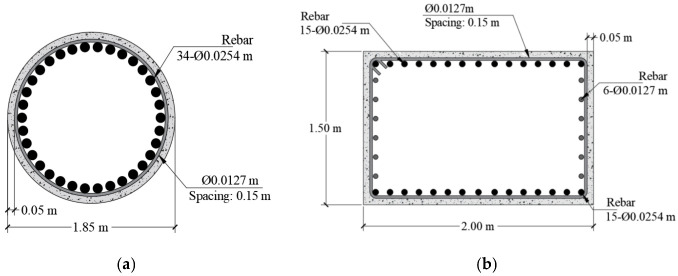
Geometry and design: (**a**) columns and (**b**) cap beams.

Fragility curves are computed for an RC bridge conceived to perform a drift equal to 0.002. The structural system consists of 4 spans with a total length of 130 m and 8 m of height clearance. A compressive strength, f’c, equal to 30 MPa, is used for columns and cap beams, while a value of 40 MPa is considered for AASHTO beams. Figure 2 shows the cross-section of the bridge, and Figure 3 shows the longitudinal view. The structure reports a fundamental period of 0.28 s. Figure 4 shows the dimensions and reinforcement of the columns and cap beam sections. 

### 5.1. Uncertainties for RC Bridges

The uncertainties related to the manufacturing and construction processes of the materials are used to calculate the potential bias that is not considered when nominal properties are used. Uncertainties have a significant influence by either increasing or decreasing the structural response. When such a response is expressed in terms of a reliability indicator, it is possible that the system presents desirable or undesirable reliability levels due to the consideration of uncertainties. Table 1 shows the mechanical uncertainties of materials; geometric uncertainties of structural sections are shown in Table 2. The uncertainties associated with structural and nonstructural elements are shown in Table 3. Table 4 and Table 5 show the parameters to estimate the corrosion initiation and cracking time, respectively.

### 5.2. Nonlinear Modelling

Structures are exposed over time to different environmental loads, such as seismic, wind, and waves, among others. If the occurrence of an environmental load is similar to the solicitation that governed the design, there is a high probability that the structure exhibits certain damage. Structural damage can be modeled numerically using two philosophies: (a) Distributed plasticity; (b) Concentrated plasticity. The distributed plasticity philosophy can be modeled by finite element techniques, while concentrated plasticity is modeled considering that plastic hinges occur close to both ends of the elements. The structure under study is modeled by means of concentrated plasticity using the Ruaumoko 3D program [60]. It is assumed that columns and cap beam elements provide the lateral stiffness of the system. Furthermore, the bridge deck only transmits dead loads. The modified Takeda hysteresis rule is used to estimate the plastic hinges. The Takeda rule is defined by different parameters, such as initial stiffness, $k_{0}$; $k_{u}$ is the stiffness in the discharge branch; r controls the loss of stiffness after yield, with values between 1 to ∞; α and β control the discharge branch with values equal to 0.5 and 0.6, respectively. The moment–curvature relation for each structural element is estimated considering the stress–strain model for confined concrete, proposed by [61] and the investigation proposed by [62] for Mexican steel reinforcement. 

### 5.3. Waiting Times and Intensities

**Figure 5:**
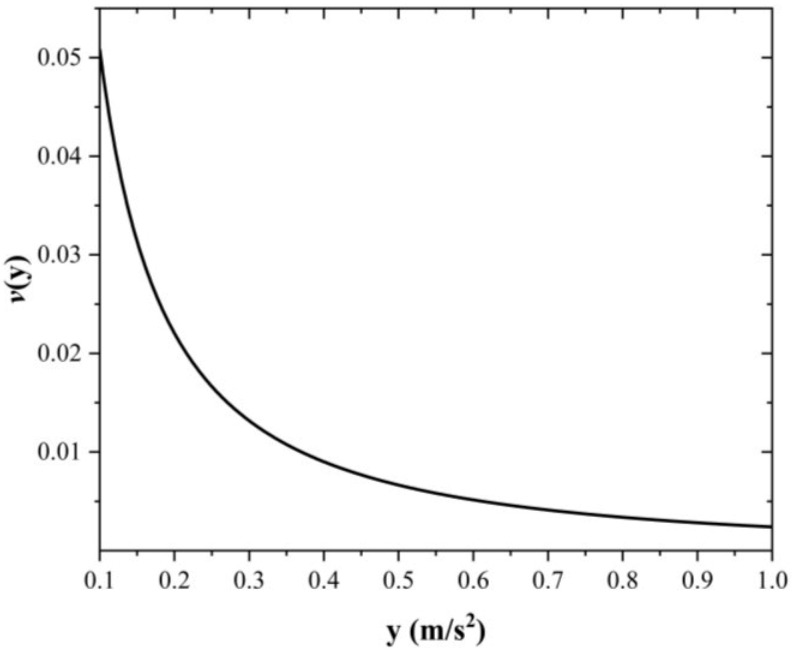
Seismic hazard curve used for the analysis, $T_{0}$ = 0.28 s.

In order to estimate the possible damage that the system could experience in a time interval, it is necessary to simulate solicitations and their occurrences. The intensities are simulated based on the seismic hazard curve (SHC) (see Figure 5) of the site, associated with the fundamental period and specific critical damping. The SHC indicates the number of times that a certain level of seismic intensity per unit of time is exceeded. Intensity simulation is made according to the cumulative distribution function, CDF, of the SHC as $F\left(y\right)=1-{\mathrm{SHC}}_{FIT}\;/v_{0}$ where ${\mathrm{SHC}}_{FIT}$ represents the fitting function as ${\mathrm{SHC}}_{FIT}={\left(y/y_{0}\right)}^{-r}{\left(y_{max}-y/y_{max}-y_{0}\right)}^{\varepsilon }$, $y_{0}$ is the seismic intensity necessary to produce damage to the structure. In this case $y_{0}=$1 m/s^2^ associated with an exceedance rate equal to $v_{0}=$0.05081; $y_{max}$ represents the last intensity of the SHC; r and ε fitted the SHC. It is assumed that seismic occurrences follow a Poisson-type process so that the waiting times between events are distributed exponentially [63]. After some mathematical steps about CDF, the time occurrence of seismic loads is $T_{i}=-\left|\ln\left(u\right)/v_{0}\right|$ where u is estimated based on a uniform distribution [13]. 

### 5.4. Seismic Loadings

**Figure 6:**
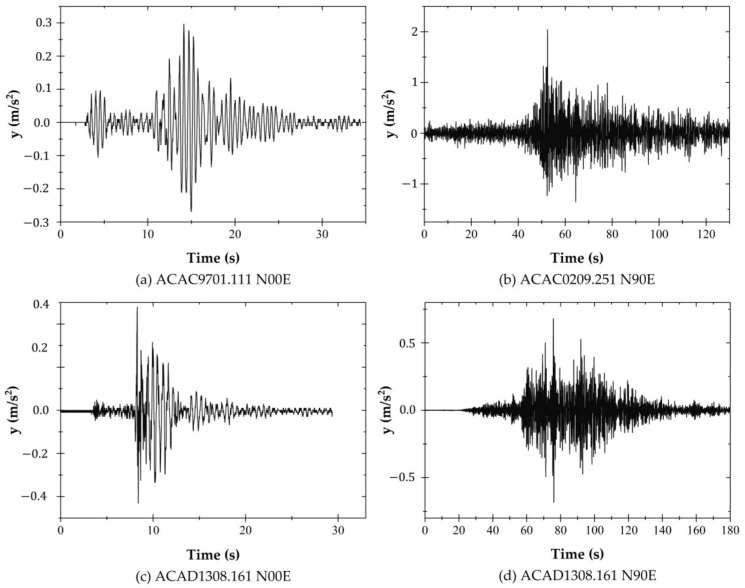
Time history data of four seismic records.

The ground motion data set used as input for the seismic simulations has been extracted from two seismographic stations close to the study case, which complies with forty-six ground motions recorded during the past several decades that are related to events with a magnitude between 4 and 7.5 [40]. The mean epicentral distance is equal to 170.58 km. The dominant period of the soil site is around 0.51 s. Figure 6 shows only four ground motions. 

### 5.5. Structural Demand over Time

**Figure 7:**
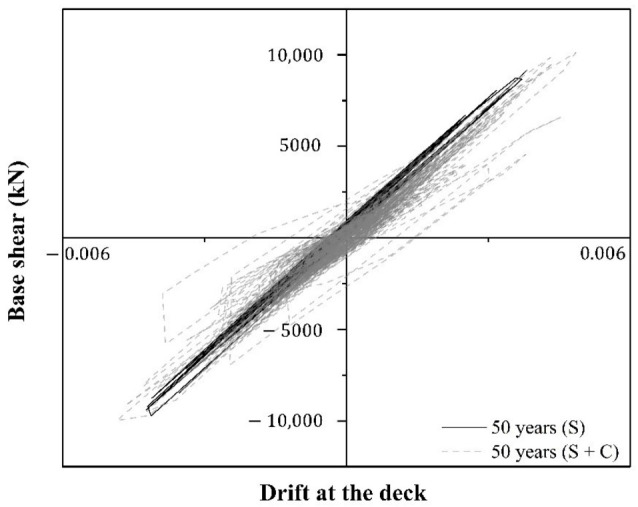
Global structural response due to seismic loads (S) and due to seismic loads plus corrosion (S + C).

The structural demand is estimated considering different time stages such as 0, 50, 75, 100, 125, and 150 years. Fifty models with mechanical and geometric properties are generated. The simulation of both such properties and nonstructural elements are based on their respective probability distribution function shown in Table 1, Table 2 and Table 3, respectively. The simulated i-th property is associated with the i-th structural model. Then, the fifty models with simulated properties present fundamental periods between 0.25 and 0.34 s. As previously mentioned, the fundamental period of the system with nominal properties is equal to 0.28 s. Therefore, the uncertainties modify both the dynamic characteristics of the system and its seismic response. The bridge is located in a tidal zone; thus, the water-to-cement ratio of concrete is 0.45 [64]. Parameters $i_{corr}$ and α are characterized by a uniform distribution function with values from 0.95 to 1.90 μA/cm^2^ [65] and 0.523 and 0.622 [53], respectively. For stages less than $T_{corr}$, the reinforcement cross-sectional area is intact. On the other hand, the area of the reinforcement bars is reduced for stages greater than $T_{corr}$ (Equation (4)). The statistical parameters shown in Table 4 and Table 5, which are associated with the i-th structural model, are used to simulate both corrosion initiation and cracking times. The mean value of corrosion initiation time, ${\hat{T}}_{corr}$, is equal to 45 years, and the mean cracking time is equal to 57 years. The 75 years threshold refers to the life span of bridges based on the AASHTO code [66]. Time thresholds of 100, 125 and 150 years are considered to observe the evolution of the corrosion deterioration. The structural demand is obtained based on the cumulative damage process described previously. One hundred realizations of waiting times and seismic intensities associated with one hundred structural models with uncertain properties are considered. The structural demand is estimated by means of nonlinear, step-by-step, dynamic analysis. Figure 7 shows an example of the global response of the system after 50 years, expressed in terms of global drift versus base shear, considering both cases: seismic sequences (S) and seismic sequences plus the effect of corrosion (S + C). At 50 years, the corrosion process has begun, and only the diameter of the steel reinforcement is reduced. It is noticed that the structural response under the effect of S + C presents both a greater reduction in stiffness and an increase in global drift compared with the case in which the system is subjected only to seismic loads S. In the case of S + C, corrosion affects the moment–curvature relationship because it is estimated considering the reduced diameter at time t, $H\left(t\right)$. Then, there is a reduction in both the yield moment and ultimate moment, which explains the differences between S versus S + C on the structural response. On the other hand, the parameters that contribute to the response of the system are earthquakes with a high magnitude whose dominant period of their response spectra is close to the dominant period of the structure. In the case of corrosion, its initiation time is reduced with high values of both critical ion concentration, $C_{cr}$, and temperature, ϕ. Cracking time is reduced in the case of either a high corrosion rate, $i_{corr}$, or low steel density, $\rho_{steel}$.

**Figure 8:**
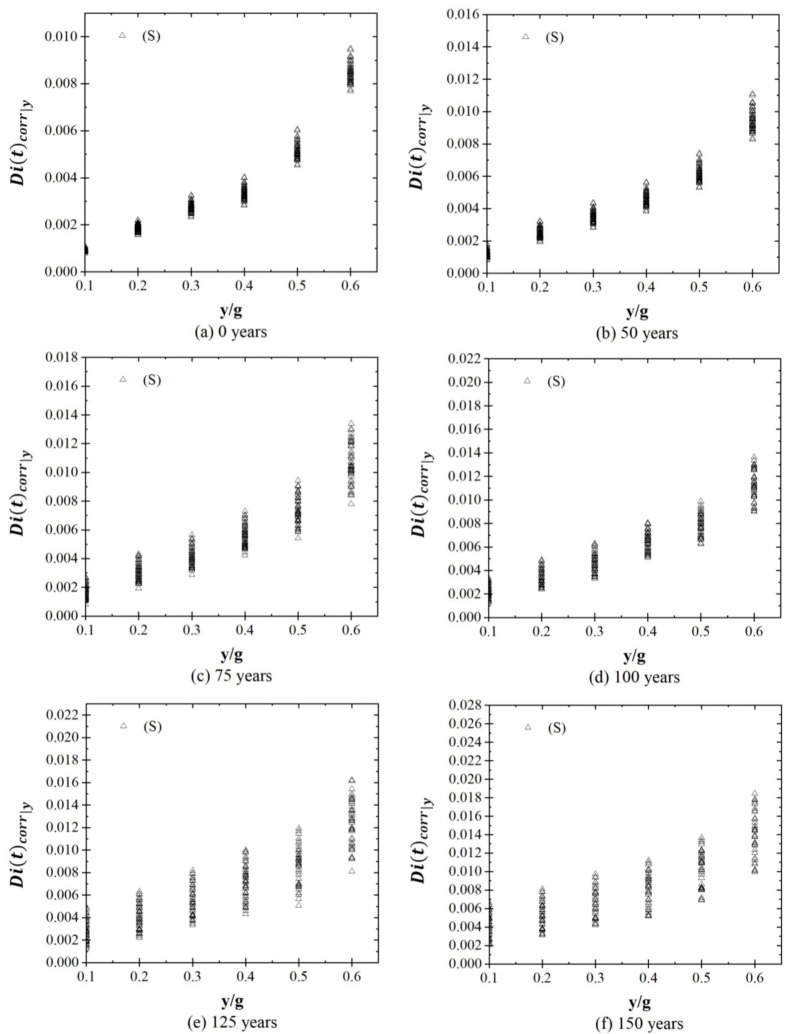
Structural response for stages due to seismic loads.

**Figure 9:**
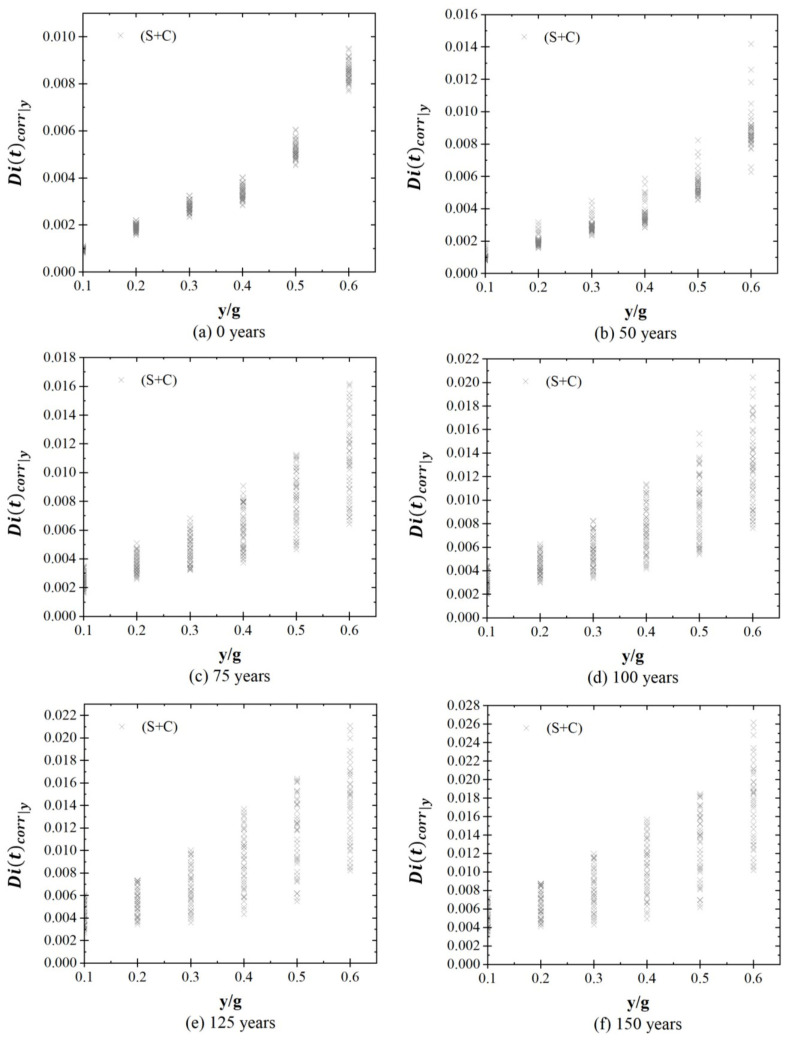
Structural response for stages due to seismic loads plus corrosion.

Figure 8 and Figure 9 show the structural response of the system,$\;Di_{corr}{}_{\left|y,t\right.}$, in terms of global drift for different values of y/g at time stages of 0, 50, 75, 100, 125, and 150 years, considering both cases (S and S + C). Figure 8 shows that the structural demand varies between 0.0008 and 0.0095 at 0 years, and values between 0.0021 to 0.0184 after 150 years are presented. Figure 9 shows that the structural response of the system varies between 0.0008 to 0.0095 at 0 years, and the structural response increases between 0.0102 and 0.0262 after 150 years of the system’s construction. 

**Figure 10:**
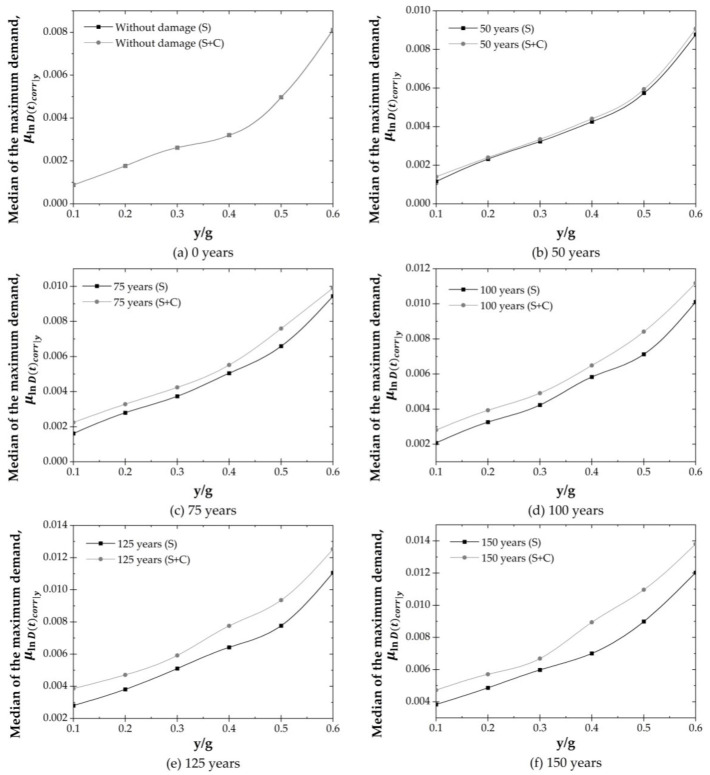
Median demand for stages of 0, 50, 75, 100, 125 and 150 years due to seismic loads (S) and due to seismic loads plus corrosion (S + C).

Figure 10 shows the median demand response due to seismic sequences (S) and seismic sequences plus the effect of corrosion (S + C). In the case of the structural demand that only considers the seismic sequences, the value of the median of the structural demand, for instance, at 0, 50, 75, 100, 125 and 150 years, associated with seismic intensities of 0.10 y/g are 0.00088, 0.00115, 0.00161, 0.0021, 0.0028 and 0.0038, respectively. This implies an increment due to earthquakes of 31.04%, 82.66%, 134.28%, 216.95% and 333.54%, respectively. When the seismic sequences plus corrosion over time is considered, it is observed that the demand value increases by about 60.33% in the stages of 50 to 75 years, associated with intensities of 0.10 y/g, due to the appearance of cracking in the concrete. On the other hand, it is shown that the initial ordinate of structural demand increases from 0 years (without damage) to 150 years by 434.49%. Table 6 shows the standard deviation of the natural logarithm of the structural response, $\sigma_{\ln D{\left(t\right)}_{corr}{}_{\left|y\right.}}$, for both cases at time stages of 0, 50, 75, 100, 125, and 150 years. It is noticed that the standard deviations increase as both y/b and time stages increase. Values of $\sigma_{\ln D{\left(t\right)}_{corr}{}_{\left|y\right.}}$ between 0.0069 and 0.161 are obtained for the case of (S), and values between 0.013 and 0.292 are estimated for the case of (S + C). 

### 5.6. Fragility Curves over Time

**Figure 11:**
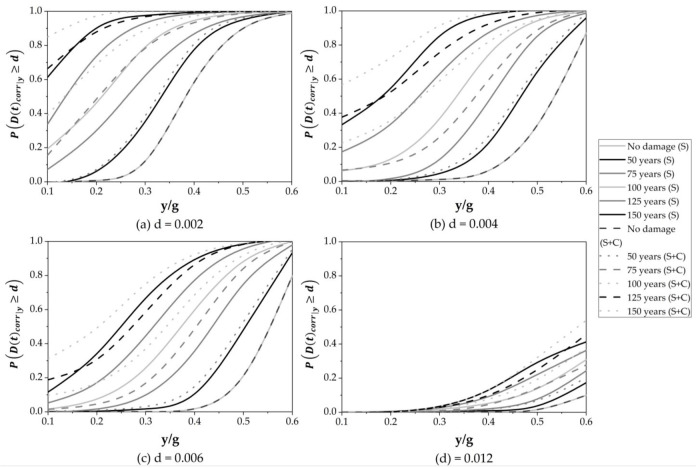
Fragility curves over time at stages of 0, 50, 75, 100, 125 and 150 years.

The vulnerability of bridges is one of the priorities of crisis management governments to plan risk reduction. Fragility curves are constructed to assess structural vulnerability due to earthquakes and corrosion. The maximum drift ratio at the bridge deck is defined as a demand parameter. Fragility curves are obtained considering four performance levels, for instance, 0.002, 0.004, 0.006 and 0.012. Figure 11 provides the following information: (1) The continuous line represents the cumulative damage due to seismic loads, and the dashed line is used to represent the results of seismic loads and corrosion deterioration over time; (2) Figure 11a shows that the probability of exceeding 0.002 is close to one for values greater than or equal to 0.6 y/g in all cases. On the other hand, the probability of exceeding 0.002 is close to zero for seismic intensities lower than 0.15 y/g in the stages of 0 and 50 years for both S and S + C. An increase in the ordinate is appreciated for stages greater than 50 years because of cumulative damage over time due to earthquakes and corrosion; (3) Figure 11b shows that the probability of exceeding the drift threshold of 0.004 is close to zero when only seismic loads associated with intensities smaller than 0.15 y/g occur from 0 to 75 years. On the other hand, intensities smaller than 0.15 y/g present probabilities close to zero for time stages of 0 and 50 years when corrosion plus earthquakes are considered; (4) Figure 11c shows that the performance level of 0.006 is reached for values greater than or equal to 0.5 y/g in the cases of 125 and 150 years when seismic loads are considered. The probability of exceeding 0.006 is close to zero for values lower than 0.20 y/g for 0 and 50 years; (5) Figure 11d demonstrates that the highest probability of exceeding the performance level of 0.012 is equal to 0.41 when the damage due to earthquakes is considered. When corrosion deterioration is not neglected, the probability of exceeding 0.012 increases to 30.51%.

## 6. Research Significance 

The manuscript presents a methodology to estimate the probability of exceeding a certain performance level considering the effect of seismic loads and seismic loads plus corrosion. Such cases modify the safety level of the structure over time. Such a change depends on the type of solicitation or any combination of them. In the case in which the structure is subjected only to seismic loads, the effect of cumulative damage is present after the time stage of 0 years. Then, the probability of exceeding each performance threshold increases, and no significant change is noticed between the involved time stages. However, the phenomenon of corrosion combined with seismic loads moderately increases the probability of exceeding each performance level after the steel reinforcement begins to deteriorate due to corrosion. Moreover, the structural system exhibits both a decrease in lateral stiffness and high deterioration of the steel reinforcement after cracking occurs. Thus, an important increment in the probability of exceeding different thresholds at the stages of time of 75, 100, 125, and 150 years is present. If the effect of corrosion plus seismic loads is considered, the probability of exceeding the design performance level equal to 0.002 increases with a mean percentage of 133% at the time stage of 75 years. Such a difference implies that $P\left(D{\left(75\right)}_{corr}{}_{\left|y\right.}\geq 0.002\right)$ is equal to 1 at y/g = 0.5. The same probability without corrosion occurs at y/g = 0.6. The above difference in terms of y/g indicates that a less intense ground motion is required to exceed the value of 0.002 for the case of seismic loads plus corrosion, which highlights the importance of the effect of corrosion in the estimation of the cumulative damage caused by seismic loads over time. 

## 7. Conclusions

A methodology was proposed to obtain fragility curves under the influence of seismic sequences and corrosion deterioration for different time stages. The methodology considers all the possible intensities that can occur on the structure as well as the uncertainties in mechanical, geometric and corrosion phases. The steps to estimate the structural fragility over time are relatively easy to use by structural engineers, but with the difficulty that it requires considerable computational time.

Fragility curves were obtained, establishing performance levels equal to 0.002, 0.004, 0.006 and 0.012, all of them associated with time stages of 75, 100, 125 and 150 years. Initial cumulative damage at y/g = 0.1 is observed for the threshold equal to 0.002 with probability values between 0.07 and 0.61, and between 0.17 and 0.85 for the time stages 75 to 125 years, considering the case in which the cumulative damage is quantified by the actions of earthquakes and earthquakes plus corrosion, respectively. Thus, it is noticed that the initial cumulative damage becomes important as time increases. The probability of exceedance 0.012 was not present in any case over time. On the contrary, the probability of exceeding the performance levels of 0.002 and 0.004 are present at 75, 100, 125, and 150 years, while the same probability and time stage is present for the drifts equal to 0.002, 0.004 and 0.006 for the case in which seismic occurrences and corrosion are considered. Based on the results obtained, and following the recommendations given by the AASHTO code, it is not recommended to design bridges to perform a drift threshold of 0.002 with similar topology and location if consideration of the effect of seismic loads and corrosion over time is required. Such a recommendation only indicates that the bridge structure under study would exceed the serviceability limit state, and it could present a certain probability of exceeding the system’s collapse after 75 years of the bridge construction. The presented results can help to understand the safety at different time stages of this kind of structure under seismic loads and corrosion. 

The methodology discussed can be helpful for decision-making on the design or re-design of new structures that can be conceived to develop a certain drift threshold. The assumptions used in the expressions to calculate the different corrosion phases lead to an approximation. Therefore, it is not possible to identify an important element, such as the crack pattern. Such shortcomings may be addressed using an anisotropic model made with numerical techniques such as the finite element method and validated by testing. Cumulative damage demands more computational time when it is estimated by means of the Monte Carlo technique. Computational time may be reduced using techniques such as Latin Hypercube Sampling. Future works may be developed based on fragility curves over time, such as demand exceedance rates, to estimate the return period that a certain performance level is exceeded. In addition, fragility curves may be incorporated to estimate the expected cost of maintenance in order to establish repair policies with the aim of extending the lifespan of the system. 

## Figures and Tables

**Table 1:** Mechanical uncertainties of materials.

Material	Nominal Resistance (MPa)	Distribution	Mean (MPa)	C.V.	Reference
Concrete	27.60	Normal	34.22	0.15	[54]
	31.00	Normal	37.21	0.14	
	41.40	Normal	47.61	0.125	
Steel reinforcement	412	Normal	448.85	0.0369	[55]

**Table 2:** Geometric uncertainties of structural elements [55].

	Distribution	Bias Factor	C.V.
Slab element	Normal	+7.62 × 10^−4^	6.60 × 10^−3^
Beam height	Normal	−5.334 × 10^−3^	6.35 × 10^−3^
Beam width	Normal	+2.54 × 10^−3^	3.81 × 10^−3^
Column dimension	Normal	+1.524 × 10^−3^	6.35 × 10^−3^
Cover	Normal	+8.128 × 10^−3^	4.318 × 10^−3^

**Table 3:** Uncertainties for structural and nonstructural elements [56].

	Distribution	Bias Factor	C.V.
Factory items	Normal	1.03	0.08
Site elements	Normal	1.05	0.10
Asphalt	Normal	0.075 *	0.25
Nonstructural elements	Normal	1.03–1.05	0.08–0.01

* mean thickness.

**Table 4:** Variables involved in the diffusion model.

Parameter	Distribution	Mean	Standard Deviation	Reference
$\mathrm{Cover},\;d_{0}$ (m)	Normal	8.128 × 10^−3^	4.318 × 10^−2^	[55]
$\mathrm{Chloride}\;\mathrm{concentration}\;\mathrm{in}\;\mathrm{the}\;\mathrm{exposed}\;\mathrm{zone},\;C_{0}$ (%)	Normal	10.918 × 10^−2^	6.56 × 10^−2^	[57]
$\mathrm{Initial}\;\mathrm{chloride}\;\mathrm{concentration},\;C_{i}$ (%)	Deterministic	0.00	-	[25]
$\mathrm{Critical}\;\mathrm{concentration}\;\mathrm{of}\;\mathrm{chloride}\;\mathrm{ions},\;C_{cr}$ (%)	Uniform	2.5 × 10^−2^	3.75 × 10^−2^	[57]
Temperature, ϕ (°C)	Normal	27.92	1.47	[58]

**Table 5:** Variables to estimate the cracking corrosion time.

Parameter	Distribution	Mean	Standard Deviation	Reference
$\mathrm{Rust}\;\mathrm{density},\;\rho_{rust}$ (ton/m^3^)	Normal	3.60	0.36	[26]
$\mathrm{Pore}\;\mathrm{cement}\;\mathrm{size},\;t_{pore}$ (mm)	Lognormal	12.5	2.54	[25]
$\mathrm{Diameter}\;\mathrm{of}\;\mathrm{rebar}\;\mathrm{for}\;\mathrm{cap}\;\mathrm{beams},\;\eta_{0beam}$ (m)	Normal	2.5 × 10^−2^	±4	[59]
$\mathrm{Diameter}\;\mathrm{of}\;\mathrm{rebar}\;\mathrm{for}\;\mathrm{cap}\;\mathrm{beams},\;\eta_{0col}$ (m)	Normal	3.2 × 10^−2^	±4	[59]
$\mathrm{Steel}\;\mathrm{density},\;\rho_{steel}$ (ton/m^3^)	Normal	8.00	0.80	[26]
$\mathrm{Poisson}\;\mathrm{ratio},\;\nu_{c}$	Deterministic	0.25	-	-

**Table 6:** Standard deviation of the natural logarithm of demand.

y/g	Seismic Loads (S)						Seismic Loads Plus Corrosion (S + C)					
	0 Years	50 Years	75 Years	100 Years	125 Years	150 Years	0 Years	50 Years	75 Years	100 Years	125 Years	150 Years
0.10	6.9 × 10^−3^	1.6 × 10^−2^	2.6 × 10^−2^	3.7 × 10^−2^	4.6 × 10^−2^	5.9 × 10^−2^	1.3 × 10^−2^	2.8 × 10^−2^	4.6 × 10^−2^	7.2 × 10^−2^	9.8 × 10^−2^	1.2 × 10^−1^
0.20	1.2 × 10^−2^	2.1 × 10^−2^	3.3 × 10^−2^	4.7 × 10^−2^	5.7 × 10^−2^	6.9 × 10^−2^	2.8 × 10^−2^	5.1 × 10^−2^	7.14 × 10^−2^	9.1 × 10^−2^	1.3 × 10^−1^	1.4 × 10^−1^
0.30	1.8 × 10^−2^	3.4 × 10^−2^	4.7 × 10^−2^	6.1 × 10^−2^	7.7 × 10^−2^	9.8 × 10^−2^	3.8 × 10^−2^	7.7 × 10^−2^	1.1 × 10^−1^	1.4 × 10^−1^	1.6 × 10^−1^	1.9 × 10^−1^
0.40	2.3 × 10^−2^	3.8 × 10^−2^	6.0 × 10^−2^	8.8 × 10^−2^	1.10 × 10^−1^	1.27 × 10^−1^	7.1 × 10^−2^	1.0 × 10^−1^	1.3 × 10^−1^	1.5 × 10^−1^	1.7 × 10^−1^	1.9 × 10^−1^
0.50	3.3 × 10^−2^	5.9 × 10^−^2	9.3 × 10^−2^	1.2 × 10^−1^	1.41 × 10^−1^	1.69 × 10^−1^	9.28 × 10^−2^	1.22 × 10^−1^	1.48 × 10^−1^	1.75 × 10^−1^	1.96 × 10^−1^	2.17 × 10^−1^
0.60	8.3 × 10^−2^	1.0 × 10^−1^	1.2 × 10^−1^	1.4 × 10^−1^	1.61 × 10^−1^	1.84 × 10^−1^	1.22 × 10^−1^	1.61 × 10^−1^	1.87 × 10^−1^	2.14 × 10^−1^	2.44 × 10^−1^	2.92 × 10^−1^

## Data Availability

The data presented in this study are available on request from the corresponding author.
